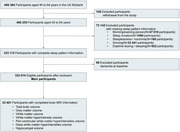# Associations of Adherence to a Healthy Sleep Pattern with the Dementia Risk in the UK Biobank

**DOI:** 10.1002/alz70860_098748

**Published:** 2025-12-23

**Authors:** Tao Wei, Jie Chang, Yi Tang

**Affiliations:** ^1^ Xuanwu Hospital, Capital Medical University, Beijing, Beijing, China; ^2^ National Center for Neurological Disorders, Xuanwu Hospital, Capital Medical University, Beijing, Beijing, China; ^3^ Department of Neurology & Innovation Center for Neurological Disorders, Xuanwu Hospital, Capital Medical University, National Center for Neurological Disorders, Beijing, Beijing, China

## Abstract

**Background:**

Existing evidence highlights associations between sleep behaviors and dementia risk; however, the impact of adhering to a healthy sleep pattern on dementia risk remains unclear.

**Method:**

Conducted as a cohort study utilizing data from UK Biobank participants aged 40–64, with complete sleep pattern information and free of dementia at baseline. Enrolment occurred between 2006 and 2010, with follow‐up until December 2022. The degree of adherence to a healthy sleep pattern was determined based on five sleep behaviors, including sleeping 7–8 hours/day, early chronotype, no frequent insomnia, no snoring, or no frequent daytime sleepiness, with a greater score indicating better adherence. Cox proportional hazards models assessed the association between a healthy sleep pattern and dementia risk.

**Result:**

A total of 3035 incident cases of dementia were documented, including 1304 Alzheimer's disease cases and 597 vascular dementia cases. An association between the adherence to a healthy sleep pattern and decreased dementia risk was observed: with each 1‐point increase in the score, the hazard ratio (HR) (0.93; 95% confidence interval [CI]: 0.89–0.96) decreased by 7.0%. Compared to participants scoring 0–1, the HR of 5‐scored dementia risk was 0.75 (0.61–0.92). Benefits were more pronounced in adults aged 40–55 years than those aged 56–64 years (*p* for interaction < 0.001). Sticking to a healthy sleep pattern was associated with increased total brain, white matter, and grey matter volumes, along with lower white matter hyperintensity (all *p* < 0.05). Mediation analysis indicates that preserving grey and white matter integrity partially mediated the dementia‐risk‐lowering benefit (*p* < 0.05).

**Conclusion:**

Following a healthy sleep pattern is linked to a reduced risk of dementia in middle‐aged adults, with the effect being particularly pronounced among those who adhere to such a pattern from earlier ages.